# Effect of Physical Training on the Morphology of Parasympathetic Atrial Ganglia after Unilateral Vagotomy in Rats

**DOI:** 10.3390/jcdd9110391

**Published:** 2022-11-13

**Authors:** Oleg V. Mamontov, Roman V. Grozov, Sarkis M. Minasian, Sergei G. Zhuravskii, Michael M. Galagudza, Alexei A. Kamshilin

**Affiliations:** 1 Almazov National Medical Research Centre, Saint Petersburg 197341, Russia; 2 Institute of Automation and Control Processes of the Far Eastern Branch of the Russian Academy of Sciences, Vladivostok 690041, Russia

**Keywords:** atrial ganglionated plexus, autonomic dysfunction, parasympathetic denervation, heart transplantation, unilateral vagotomy, heart rate variability, immunohistochemical study

## Abstract

Cardiac denervation is a serious problem in a number of patients, including patients after heart transplantation. The status of the parasympathetic ganglia after crossing the preganglionic fibers of the vagus nerve has not been enough studied. The aim of our study was to assess the effect of physical training on the morphological parameters of the parasympathetic atrial ganglia and autonomic regulation of heart rate after right- and left-sided vagotomy in rats. Morphometric characteristics of the right atrial ganglia were evaluated using an immunohistochemical method after a study that included a three-time assessment of heart rate variability. It was found that right-sided vagotomy leads to both an increase in the volume of ganglion and autonomic dysfunction. No significant change in the number of nerve cells was found in animals with false and left-sided vagotomy while maintaining preganglionic innervation after the physical training, whereas exercises led to a decrease in the volume of nerve tissue of rats with right-sided denervation. It was also found that in animals with preserved vagal innervation, the volume of atrial ganglion tissue correlates with overall heart rate variability and a normalized parasympathetic component. Therefore, a positive effect from regular physical activity on parasympathetic regulation can be expected only if preganglionic vagal influence is preserved.

## 1. Introduction

In healthy individuals, heart rate (HR) is continuously adjusted to highly variable levels of physical activity by complex regulatory mechanisms [1]. It has been suggested that the trophic effects of the vagus nerve on the myocardium are of great importance for maintaining the energy metabolism of cardiomyocytes, which ultimately affects exercise tolerance [2]. It is well established that the loss of cardiac parasympathetic innervation leads to a persistent increase in HR, which negatively affects both the diastolic function of the myocardium and its metabolism [3,4]. Apparently, vagal effects on the heart, which begin to increase immediately after cessation of physical exercise, are very important for ensuring the overall physical performance of the organism [5,6]. Various alterations of parasympathetic effects on the heart may be used as predictors of unfavorable outcomes in patients with cardiovascular diseases [7]. Such disorders are anticipated to be maximal in patients with cardiac transplantation, as the transplanted heart is at least temporarily subjected to complete autonomic denervation, which contributes to postoperative complications related to both the control of blood pressure (BP) and exercise tolerance [3,8]. 

Currently, data on the extent of cardiac autonomic re-innervation after transplantation remains controversial. The intensity of the re-innervation may be different for the sympathetic and parasympathetic nervous system. While partial sympathetic re-innervation has been convincingly demonstrated in the late period after transplantation [9,10], the evidence for the restoration of cardiac control by the vagus nerve is less solid [11,12,13]. Consequently, a number of clinically relevant issues currently remain unresolved, such as whether some extent of parasympathetic control over the HR is still operative due to the activity of the neurons within the atrial parasympathetic ganglia after transection of preganglionic fibers running within the vagus nerve. Is regular mild physical training, which is widely recommended for patients with chronic heart failure [14,15], including those after heart transplantation [16,17], able to restore parasympathetic regulation of the vagotomized heart? In addition, there is no coherent view on the functional consequences of unilateral vagotomy. In other words, the consequences of isolated left-sided or right-sided vagal denervation in terms of heart function deserve further investigation. Some reports showed that the right-sided vagotomy resulted in a more significant alteration in the regulation of sinoatrial node activity [18,19]. However, it remains unclear to what extent this asymmetry affects the structure and function of the parasympathetic atrial ganglia. Finally, the morphological changes in atrial parasympathetic ganglia in response to transection of preganglionic fibers, which is an inevitable consequence of orthotopic heart transplantation remains to be explored.

Recently, we performed a study aiming to assess the effect of exercise training on cardiac autonomic control and exercise tolerance in rats after unilateral vagotomy [20]. In this study, the duration of running and the total distance were assessed by means of a treadmill test. In addition, the autonomic control of the heart was evaluated by assessing the heart rate variability (HRV) both before and after either left-sided or right-sided vagotomy. Vagotomized rats were randomized into two groups according to physical activity, i.e., intensive exercise training versus no exercise. It was shown that the right-sided vagotomy caused significant decrease in both exercise tolerance and HRV in non-exercising rats, whereas regular training contributed to an increased exercise tolerance after right-sided vagotomy. After the final assessment of exercise tolerance and HRV, the animals were withdrawn from the study, and their hearts were subjected to morphological evaluation particularly focused on right atrial parasympathetic ganglia.

Here, we continued the study on the base of the same biomaterial, and aimed to assess the effect of physical training on the morphology of the atrial parasympathetic ganglia, and evaluate its relationship with heart rate regulation and exercise tolerance in rats after unilateral vagotomy.

## 2. Materials and Methods

### 2.1. Animals and Ethical Aspects

All experiments were carried out in accordance with the Guide for the Care and Use of Laboratory Animals (NIH publication No. 85-23, revised 1996) and the European Convention for the Protection of Vertebrate Animals used for Experimental and other Scientific Purposes, as well as the ARRIVE 2.0 guidelines. The Institutional Animal Care and Use Committee at the Almazov National Medical Research Centre approved the study protocol (Protocol Number 21-11PZ#V2; 11 June 2021). All efforts were made to protect the animals and minimize their suffering during the study.

Here we analyzed the morphology of the hearts explanted from the animals investigated in our preceding study [20]. The experiments were performed on 60 male Wistar rats obtained from the breeding facility of the Almazov National Medical Research Centre (St-Petersburg, Russian Federation). The weight of the animals varied from 250 to 320 g at the beginning of the experiments. Rats were housed in individually ventilated cages in groups of three in a light- and temperature-controlled environment for at least seven days prior to start of the experiment, with free access to food and water.

### 2.2. Experimental Design

Sixty rats entered the study to be distributed into one of six groups (*n* = 10 in each, Table 1): (1) control non-trained animals (CS) underwent sham surgery and were maintained on a regular basis; (2) control trained (CT) were subjected to sham surgery and physical training post-surgery; (3) left vagotomy + no training (LS); (4) left vagotomy + training (LT); (5) right vagotomy + no training (RS); (6) right vagotomy + training (RT).

The allocation of animals into groups was performed with reference to the baseline exercise tolerance. For that purpose, all rats at baseline were ranked according to the distance traveled along the treadmill moving track. When distributing the animals into six groups, a general list was taken as a basis, in which the rats were arranged according to the distance traveled, but with a variation in the order of distribution: first from the first to the sixth, and then in reverse order-from the twelfth to the seventh, then again in direct order-from the thirteenth to the eighteenth, and so on. From this list, the rats with numbers 1, 7, 13, 19, 25, 31, 37, 43, 49, 55 constituted the first group, the rats numbered as 2, 8, 14, 20, 26, 32, 38, 44, 50, 56-the second, etc. With such a distribution by groups, the average baseline indicators of tolerance of animals to physical activity in different groups did not differ either in the distance traveled (F = 0.006, *p* = 0.99) or in the duration of the load (F = 0.031, *p* = 0.99). One animal was excluded from the RS group due to death on the second day after surgery.

### 2.3. Assessment of Rapid Changes in Heart Rate

Using the ECG data of the animals, we calculated an index characterizing the most rapid changes in heart rhythm: the standard deviation of the normal heart rate intervals (SDNN, ms). This parameter is associated with the magnitude of sinus arrhythmia, allowing us to evaluate the influence of the parasympathetic system on HR [21,22]. SDNN was used to explore the relationships between morphometric parameters and autonomic regulation.

### 2.4. Unilateral Vagotomy

Cardiac denervation surgery was performed according to the protocol described in detail in our previous study [20]. Briefly, after general anesthesia with a mixture of Zoletil 100 and Xylazine 2%, one of three types of surgery was performed on spontaneously breathing animals: (1) left-sided transection of the vagosympathetic trunk, (2) right-sided transection, or (3) sham procedure, in which the vagosympathetic trunk was isolated and taken to the holders, but transection was not performed. After a longitudinal incision of the skin on the anterior surface of the neck, we mobilized the mandibular gland, exposing rat’s fascia and neck muscles, after opening which and displacing the sternocleidomastoid muscle, the neurovascular bundle containing the vagosympathetic trunk and the common carotid artery was exposed. When the vagus was isolated, it was taken on holders, and then either a fragment of 2–3 mm was cut off during the true vagotomy, or the vagus was placed back with a false operation. The differences in left-sided and right-sided vagotomy consisted in the choice of the side of access to the vagus nerve. Before and after any type of surgery, an electrocardiogram (ECG) was recorded for subsequent HRV assessment. After complete wound healing and training groups were subjected to intensive physical exercise on treadmill for two weeks (groups CT, LT, and RT in Table 1), while no training groups were housed without treadmill training (groups CS, LS, and RS in Table 1). Three weeks after surgery, a final assessment of exercise tolerance and HRV was performed, after which the animals were euthanized with CO_2_ and their hearts were harvested for subsequent histological and immunohistochemical examination.

### 2.5. Heart Explantation, Histological and Immunohistochemical Examination

Under deep anesthesia, the heart was exposed via wide bilateral thoracotomy. Prior to heart excision, a 10% solution of potassium chloride at a volume of 3 mL was injected intravenously in order to cause diastolic heart arrest. The cessation of electrical and mechanical activity of the heart was ascertained both visually and by ECG, after which the heart was immediately excised and rinsed in 0.9% sodium chloride. For histological analysis, we used right atrial tissues fixed for two days in 10% neutral (pH 7.4) paraformaldehyde in a phosphate buffer (Biovitrum, Russia). Thereafter, the tissue was dehydrated in a series of alcohols of increasing concentration and poured into paraffin blocks according to the standard histological technique. Histological slices with a thickness of 2.5–5.0 μm were prepared for further morphological examination.

**Figure 1:**
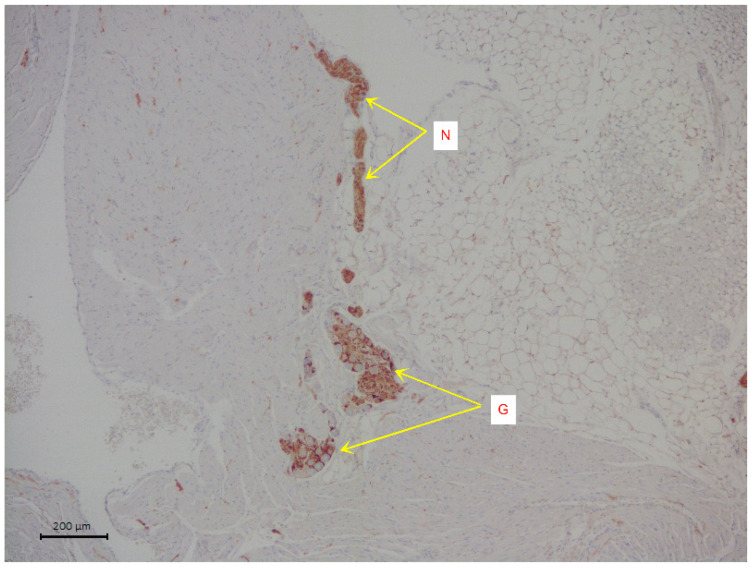
A representative image of intramural neural structures of rat right atrium. The arrows indicate parasympathetic nerve ganglion (G) and parasympathetic neural fibers (N). Immunohistochemical analysis on S100 b.

The slices were stained with hematoxylin and eosin. Immunohistochemical analysis was used to visualize the nervous tissue elements of the atrium. The cells of intramural parasympathetic ganglia were determined using staining with rabbit recombinant antibodies against the protein S100 beta [EP1576Y], a marker of astrocytes ab52642 (Abcam, Cambridge, UK). Incubation with S100 beta antibodies was carried out at a dilution of 1:2000 in a Leica Autostainer 720 during 30 min using the Novolink Max Polymer Detection system. The diaminobenzidine Novolink Max Polymer Detection System (LEICA Biosystems, Deer Park, Illinois, USA) was used to visualize the antibody fixation loci (see Figure 1). The slices were additionally stained with Mayer hematoxylin in the Leica Stainer.

### 2.6. Morphometric Analysis of Atrial Ganglia

We evaluated all intramural ganglia on digital images of slices of total specimens of the right atrium. The number of S100 b-negative cells in the tissue locus positively labeled for astrocytic protein was calculated. The average area of ganglia and the number of nerve cells per unit area (in µm^2^) were estimated. Morphometric parameters of each animal were evaluated using five histological specimens. Every fifth slice from 20 slices of the entire ganglion was evaluated. Both the number of nerve cells in the atrial ganglia and the area of ganglia were assessed as the mean over all five slices. The images were processed and morphometrically evaluated using image analysis software (NIS-Elements BR, V. 4.51, Nikon, Japan). Histological and morphometric analysis was performed using an optical microscope (Nikon ECLIPSE Ni-U, Nikon, Japan) coupled with digital camera (Nikon DS-Fi2, Nikon, Japan) at 40× or 100×. The slides were analyzed by a pathologist blinded to the treatment mode used for each group.

### 2.7. Statistical Analysis

All data are presented as mean ± standard deviation. Morphometric data are presented as box plots with median and quartiles; whiskers denote minimal and maximal values. Given the relatively small sample sizes, we have checked the distribution for normality. It was shown that normal distribution was not observed in all groups. For this reason, nonparametric statistical methods were used. Nonparametric Mann-Whitney, Kolmogorov-Smirnov criteria, and analysis of variance (ANOVA) were used to determine differences in the variables measured. *p* values < 0.05 were considered statistically significant.

## 3. Results

### 3.1. Identification of Parasympathetic Ganglia

**Figure 2:**
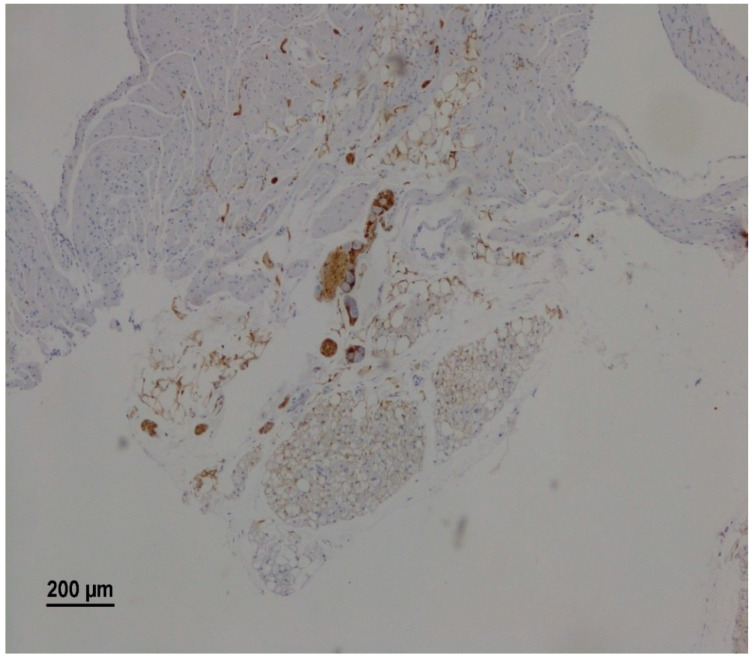
A representative microscopy image showing intramural parasympathetic ganglion. The right atrium of a rat from the RS group on the 25th day after vagotomy. Immunohistochemical staining for S100 b.

Morphological analysis identified atrial parasympathetic ganglia in 46 animals (76.3%). The neurons within the parasympathetic ganglia were readily visualized as large, neutral cells with a round nucleus, typically containing two nucleoli against the background of S100 b positive ganglionic structures such as neuroglial cells and nerve fibers (Figure 2). 

We were unable to detect the presence of ganglionic cells in atrial slices in 14 animals (23.7%). Since low variability was associated with a very small number of ganglia cells, it is possible that the absence of ganglia is an extreme degree of decrease in cell count, when a standard morphometric assessment does not reveal the presence of nerve cells. The reasons for the decrease in cellularity can vary, including hereditary characteristics of animals, as well as the influence of repeated anesthesia and inevitable stress, but this remains unclear and requires additional analysis. The number of nerve cells in the ganglia of animals ranged from 0 to 830 with an average number of cells per animal equal to 202 ± 206. The total area of the ganglia, calculated as the sum of the areas of all 5 slices, ranged from 0 to 1.55 mm^2^, with the average value for the entire sample of 0.30 ± 0.33 mm^2^. It was revealed that the number of nerve cells in the whole group (*n* = 59) strongly correlated with the total area occupied by the ganglion (r = 0.95; *p* < 0.0001).

### 3.2. Morphometric Parameters of Parasympathetic Ganglia

**Figure 3:**
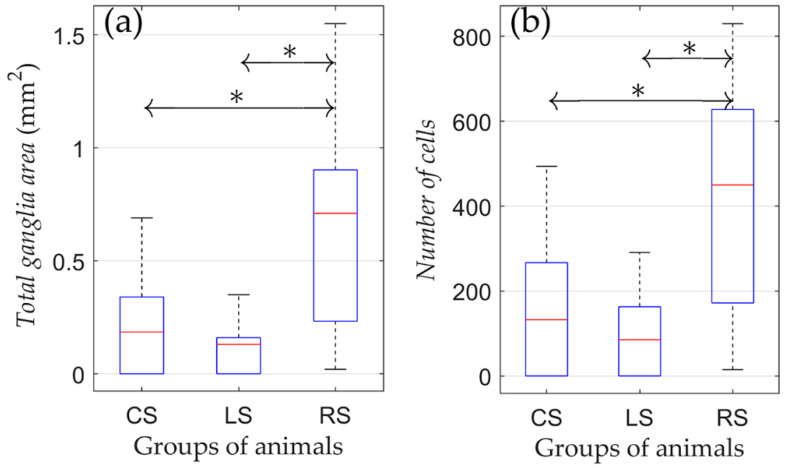
Morphometric parameters of rats that were kept in conditions of natural maintenance after the surgery (no training). (**a**) Total ganglia area and (**b**) number of cells. On each box, the central mark indicates the median, and the bottom and top edges of the box indicate the 25th and 75th percentiles, respectively. The whiskers extend to the most extreme data points not considered outliers. *: *p* < 0.05.

According to the results of the one-way ANOVA, it was found that both the number of cells and ganglion area were different among CS, LS, and RS groups (Figure 3).

The pairwise comparison revealed that cell numbers and ganglion areas between the CS and LS group were not different. Nevertheless, cell numbers and ganglion areas were both higher in the RS group compared to the CS and LS groups (*p* = 0.036 and *p* = 0.039 for the total ganglia area and number of cells, respectively). Therefore, the right-sided vagotomy resulted in an increased number of neural cells in the atrial tissue compared with the control group, whereas after left-sided denervation, there was no significant difference in these parameters. 

### 3.3. Effects of Physical Training on Morphometric Characteristics of Atrial Ganglionic Tissue

**Figure 4:**
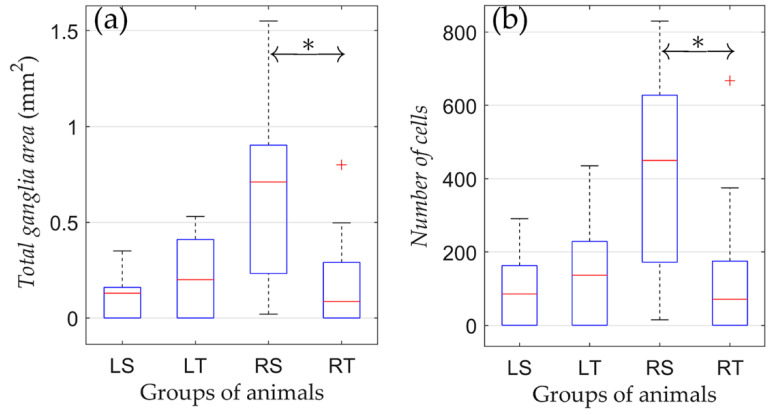
Changes in morphometric parameters due to training in rats from groups of left-sided and right-sided vagotomy. (**a**) Total ganglia area and (**b**) number of cells. On each box, the central mark indicates the median, and the bottom and top edges of the box indicate the 25th and 75th percentiles, respectively. The whiskers extend to the most extreme data points not considered outliers, and the outliers are plotted individually using the ‘+’ symbol. *: *p* < 0.05.

The effect of physical training on the state of the parasympathetic ganglia of the right atrium after various types of denervation was studied by comparing morphometric indicators in animals subjected to physical training compared to non-trained animals. No significant difference in morphometric indicators was found between trained and non-trained groups with left-sided vagotomy (LS and LT; Figure 4).

Unlike the LS and LT groups, physical training of the RT group resulted in a lower number of neural cells. In animals subjected to regular training (RT), there was a significantly reduced area of ganglionic tissue compared to the RS group (0.22 ± 0.28 vs. 0.64 ± 0.50 mm^2^; *p* = 0.04). Moreover, in the RT group, there was a trend towards a decrease in the number of nerve cells versus the RS group (166 ± 224 vs. 404 ± 292, respectively, *p* = 0.052). Therefore, the training process in animals with right-sided vagotomy was associated with a decrease in the volume of nerve tissue of the parasympathetic ganglia of the right atrium.

### 3.4. Relationship of Morphometric Characteristics with Parameters of Autonomous Regulation of Blood Circulation

**Figure 5:**
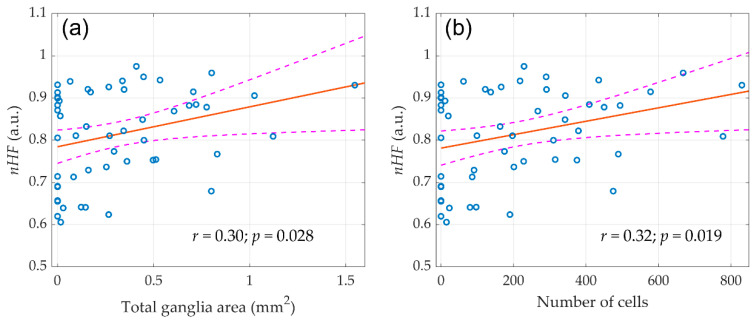
The linear regression between the initial (before any surgery) normalized high-frequency component (nHF) of the HRV spectra and morphological parameters of parasympathetic atrial ganglia. (**a**) Total ganglia area and (**b**) Number of cells. Blue circles show the measured values, the red line is the best fit for the data, and pink dashed curves represent a 95% confidence interval for the regression line.

As it was found in our previous study [20], only right-sided vagotomy has led to a significant decrease in the spectral characteristics of HRV. This reduction was evident already within the first minutes after the right vagus nerve transection, and became more pronounced three weeks after the surgery. It is noteworthy that no significant decrease in HRV was found after false and left-sided vagotomy. In this study, we revealed that despite the decrease in autonomic control after right-sided denervation, the volume of atrial ganglion tissue did not decrease. Therefore, it is of interest whether there is an association between the morphometric parameters of the ganglia and autonomic HR control evaluated by HRV. Scatter plots of the morphological parameters of the ganglia assessed in this study versus normalized high-frequency component (nHF, the frequency band of 0.8–2.5 Hz) measured in our preceding study [20] in the entire group of the animals (*n* = 59) before any surgery is shown in Figure 5.

A correlation analysis revealed that nHF positively correlated with both the number of nerve cells in the atrial ganglia (r = 0.30; *p* = 0.028) and the total area of ganglionic tissue (r = 0.32; *p* = 0.019). At the same time, both total ganglia area (r = −0,29; *p* = 0.033) and number of cells (r = −0.28; *p* = 0.035) negatively correlated with the normalized low-frequency component nLF (frequency band 0.2–0.8 Hz) of the HRV spectrum. This correlation is expected, since the nHF and nLF are inversely proportional to each other [22]. These parameters indicate the contribution of sympathetic and parasympathetic heart rate reactivity and generally reflect the dominance of one or another portion of the autonomic nervous system [23]. In other words, a larger volume of parasympathetic nervous tissue provided a greater ratio of the respiratory component in the HRV spectrum.

**Figure 6:**
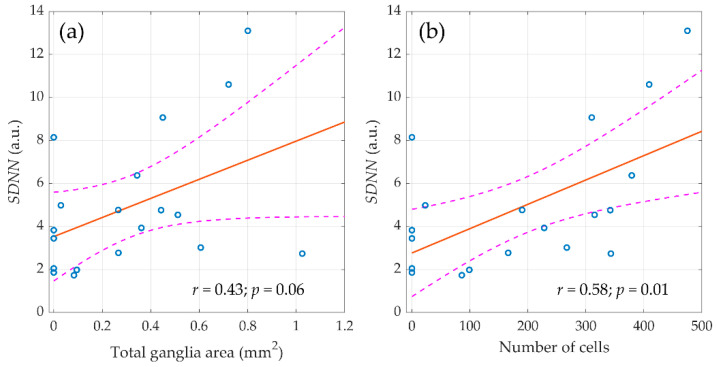
The linear regression between the standard deviation of normal RR intervals (SDNN), assessed three weeks after surgery and morphometric parameters of parasympathetic atrial ganglia. (**a**) Total ganglia area and (**b**) number of cells. Blue circles show the measured values, the red line is the best fit for the data, and pink dashed curves represent a 95% confidence interval for the regression line.

It should be noted that in the entire sample of animals (*n* = 59), the relationship of morphometric indicators was revealed only with the components of the HRV spectrum measured before surgery. However, the destruction of the autonomic regulation circuit during surgery greatly diminished the correlation between the morphological parameters of ganglion tissue and HRV. Nevertheless, in the control group (*n* = 20), the relationship between morphometric indicators and autonomic regulation persisted in the late postoperative period as shown in Figure 6. It was found that the SDNN measured in the final stage of the experiment (three weeks after surgery) correlated with the area of nerve tissue in the atrial ganglia (r = 0.44; *p* = 0.06) and with the number of neural cells (r = 0.58; *p* = 0.01).

Therefore, in animals with preserved preganglionic part of the parasympathetic nervous system, there was a direct relationship between the magnitude of autonomic influences on the sinus node at rest and the volume of ganglionic tissue, whereas the vagotomy has led not only to a decrease in rhythm variability, but also to the loss of its connection with the morphological substrate.

## 4. Discussion

In this study, we tested the hypothesis that unilateral vagotomy might affect the morphological parameters of atrial parasympathetic ganglia in rats, a process further modulated by the extent of physical activity. Our study showed that morphometric parameters, such as total ganglion area and the number of neural cells, correlated with each other. It is worth noting that morphometric indicators varied significantly from animal to animal, even in the control group. Similarly, a significant variability was observed in HRV indicators. Nevertheless, intergroup differences were revealed depending on the type of surgery. While no significant difference in ganglion morphology has been found between the animals subjected to left-sided vagotomy and sham-operated animals, it was found that the number of neural elements unexpectedly turned out to be significantly greater in animals experiencing right-sided vagotomy. However, it is well known that atrial parasympathetic ganglia are predominantly receiving input from the right vagus nerve [24,25], which is in line with our earlier observations showing that the right-sided denervation is accompanied by a decrease in HRV indices both in the early and late postoperative period [20].

At a first glance, the results obtained in this study contradict the results obtained earlier. However, such a conclusion is only reached if one does not take into account the leading role of the vagus nerve, which makes the greatest contribution to the regulation of heart rhythm [18,19], whereas the role of the sympathetic nervous system is much more modest. Our study has shown that despite the cessation of the central neural control over the heart rhythm due to vagal denervation, no involution of the intracardiac parasympathetic ganglia has occurred. On the contrary, a morphological examination indicated an increase in the volume of ganglionic tissue after right-sided vagal denervation as compared with both false and left-sided vagotomy. We hypothesized that this is due to the engagement of compensatory mechanisms restoring neural regulation of HR in conditions associated with the loss of autonomic control of HR, similarly to the changes previously observed in rabbits after vagotomy [26]. Therefore, the data obtained showed that vagal denervation does not necessarily result in the complete disappearance of all intra-organ parasympathetic ganglia containing the cell bodies of postganglionic neurons. These results give us hope that, with appropriate technology or pharmacology development, the possibility of restoring parasympathetic control after damage to the vagus nerve or heart transplantation might be envisaged in the future [27].

It is worth noting that the physical training of rats with right-sided vagotomy led to a decrease in parasympathetic indicators of the right atrium, whereas no significant changes in these parameters was observed after training of animals with either left-sided or false vagotomy. It is well known that the regular physical activity positively affects the parasympathetic nervous system [28]. Nevertheless, such an effect of physical training cannot be expected in the absence of vagal influence on the atrial ganglion, which is typical for patients after heart transplantation.

Another important observation of this study is the revealed relationship between the morphometric characteristics of the atrial parasympathetic ganglia and autonomous regulation. It is noteworthy that the area and number of neural cells were related both to the SDNN index and to the high-frequency component of the HRV spectrum (nHF) associated with parasympathetic effects on heart rate. It should be underlined that in the entire sample of animals (*n* = 59), the quantitative parameters of parasympathetic ganglia were associated only with the initial HRV indicators assessed in the preoperative period, whereas in the postoperative period, such a relationship was not observed in the entire sample. Nevertheless, in animals from the control group, the correlation between morphometric indicators and HRV persisted in the postoperative period, which is natural, since neurogenic parasympathetic control in the postoperative period was not altered in this group.

## 5. Conclusions

Our study showed that the transection of the preganglionic parasympathetic fibers within the vagus nerve does not lead to the disappearance of the ganglionic nervous tissue in the atria. Right-sided vagotomy was accompanied by an increase in the volume of ganglionic tissue, most likely due to compensatory mechanisms. In animals with impaired preganglionic innervation, a decrease in the volume of parasympathetic atrial ganglia was observed after a course of physical training. The volume of atrial ganglion tissue correlated with the overall variability of heart rate and normalized parasympathetic component in animals with preserved vagal innervation.

## Figures and Tables

**Table 1:** Abbreviated designations of the animal groups.

Life Style	Control (Sham Vagotomy)	Left-Sided Vagotomy	Right-Sided Vagotomy
No training	CS	LS	RS
Training	CT	LT	RT

## Data Availability

Not applicable.
